# Supplementary material for: Crossover from positive to negative optical torque in mesoscale optical matter
Source: Nat Commun. 2018 Nov 20;9:4897. doi: 10.1038/s41467-018-07376-7 (PMC6244235; doi:10.1038/s41467-018-07376-7)
Supplement: Supplementary file 1 — Supplementary Information [file 41467_2018_7376_MOESM1_ESM.pdf]

Supplementary Information for

Crossover from positive to negative optical torque in mesoscale optical matter

Han et al.

Supplementary Note 1: Self-organization and structural transition of optical matter isomers

Using the optical trapping system illustrated in Supplementary Figure 1, we have observed many isomers of optical matter clusters as shown in Supplementary Figure 2a. For clusters with a certain number of particles, we define the configuration that appears more often as the type-I, and other configurations are usually less stable. To understand the trend of light-mediated self-organization, we plot the sequence of adding additional NPs for the type-I structures in Supplementary Figure 2b, and rings with radii R1 to R4 that represent the first to fourth nearest neighbor distances from the first NP. A direct observation is that the NPs tend to fill up small rings first (Supplementary Figure 2b), yet for a trimer or a tetramer the new particle can also occupy position 5 on a R1 ring, leading to 3-II or 4-II rather than the type I arrays. We find that the priority of forming a certain array depends on its number of nearest neighbor pairs; an array is more likely to form when it has more nearest neighbor pairs with shorter (i.e., optical binding) distances. This trend can be seen from comparison of theoretical analysis and experimental results shown in Supplementary Figure 3. For example, the 3-particle structure 3-I has three R1 pairs while 3-II only has two, so 3-I appears more often in the data.

Therefore, we propose a rule that the priority of forming a certain array depends on its number of nearest neighbor pairs; an array is more likely to form when it has more nearest neighbor pairs with shorter distances. The statistics for isomers with 3 to 8 NPs agree well with this rule as shown in Table S1. The isomers with 9 NPs, however, have two exceptions, 9-II vs. 9-III and 9-IV vs. 9-V. From the count of pair interactions, we would expect more 9-III than 9-II and more 9-V than 9-IV, but the experimental results are the opposite. A possible reason is that 9-III can easily convert to 9-I by just moving one particle one lattice site while 9-IV can also form directly from 9-I (see Supplementary Figure 6h), so the complexity or simplicity of the dynamic transitions between these structures strongly affect the probability of observing 9-III and 9-IV configurations.

The rule is supported by the optical binding potentials. Supplementary Figure 4a shows that calculated effective optical binding potential energies between two NPs at different interparticle separations. The effective potential barrier height decays as $1/d$ with the interparticle distance d. We thus believe that the trend of forming optical matter isomers - an array is more likely to form when it has more nearest neighbor pairs with shorter distances - is related to the long-range and mutual interaction nature of the optical binding. A given NP tends to occupy a lattice site with the largest number of nearest neighbor interactions in an optical matter array, making the most stable type-I arrays least extended.

Since our probability density functions derived from long duration measurements and many samples are for steady-state conditions, we can estimate the experimental optical binding potentials in terms of the potential of mean force (pmf) using the distributions of separations between first nearest neighbors in the optical matter arrays (Supplementary Figure 4b). The pmf is determined from the measured probability density functions:

$$\mathrm{pmf}(d) = -k_{\mathrm{B}} T \ln P(d), \quad (10)$$

where k_B is Boltzmann's constant, T is absolute temperature and P(d) is the probability density as a function of the separation. For each NP the thermal energy in each dimension (degree of freedom) is $0.5k_BT$, so for two interacting NPs moving in a two-dimensional field, the thermal energy will be $2k_BT$, which is smaller than the optical binding potentials (Supplementary Figure 4c).

The optical matter isomers can change their structures during rotation. In particular, the rotation of arrays 5-I and 6-I are often associated with a single NP hopping to a nearby vacancy without moving other particles as illustrated in Supplementary Figure 5. The NP could also jump back to

the previous position due to Brownian motion. These behaviors made arrays 5-I and 6-I less stable and hence their rotational motion was not as continuously as 7-I, which does not have any vacancy that is thermally accessible. This might be a reason that the observed velocities of 5-I and 6-I are smaller than that of 7-I in Fig. 2d of the main text.

Many optical matter isomers can appear simply by relocating one NP to its first or second nearest position in a hexagonal lattice (Supplementary Figure 6a−c). The special optical matter clusters can also appear by moving two NPs simultaneously as indicated in Supplementary Figure 6d,e. It is worth noting that in Supplementary Figure 6f−h, relocation of one particle reversed the rotation direction from clockwise for array 9-I to counterclockwise for array 9-III. The array 9-IV also rotated counterclockwise while 9-II rotated clockwise.

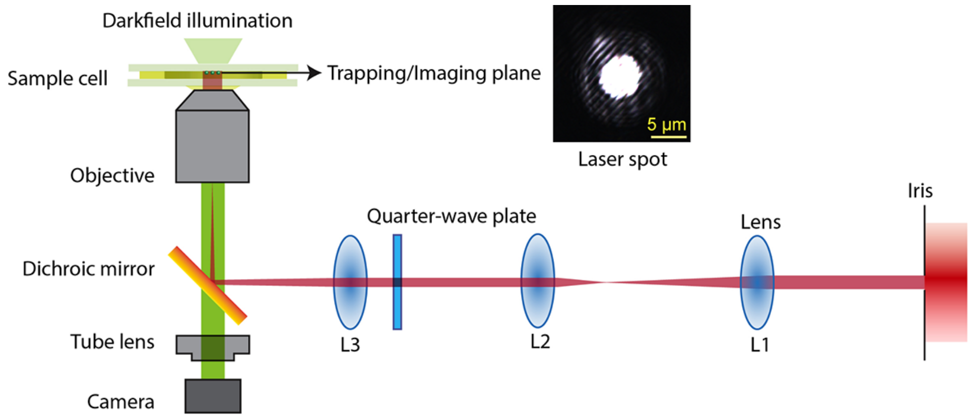

Supplementary Figure 1. Illustration of the optical trapping system used for assembling the optical matter arrays. The output from a CW Tunable Ti:Sapphire Laser (Spectra-Physics 3900S) operating at 800 nm was expanded and collimated to a Gaussian beam with ($1/e^2$) diameter of 13.5 mm. The central area (d = 2.7 mm) of the beam was selected by an iris, then collimated and reduced by a telescope with two lenses of f = 75 cm (L1) and 15 cm (L2), and finally focused by a f = 50 cm lens (L3) to the back aperture of a 60X objective (NA=1.2, Olympus UPLSAPO 60XW). The laser power was 91 mW after the iris. The image of the laser spot was taken by placing a mirror at the trapping plane and replacing the (short-pass) dichromic mirror with a 50-50 beam splitter. The interference fringes on the image are imaging artifacts, introduced by a protective glass layer in front of the camera sensor.

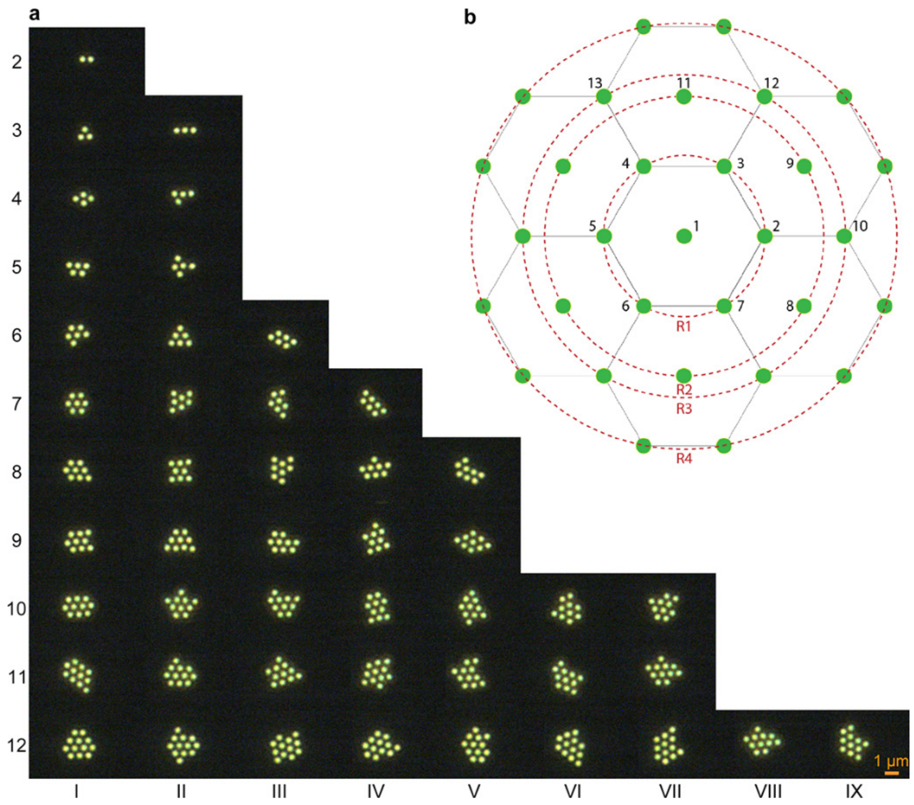

Supplementary Figure 2. Dark-field optical images and analysis of optical matter isomers. **a,** Various isomers observed in experiments. **b,** Schematic of a hexagonal lattice with preferred positions of individual NPs in the representative optical matter arrays. R1 to R4 denote the first to fourth nearest neighbor distances from the first particle. The numbering in b is both bookkeeping of particles adding to the array as well as indicating a rough priority of structures formed in building larger and larger optical matter arrays.

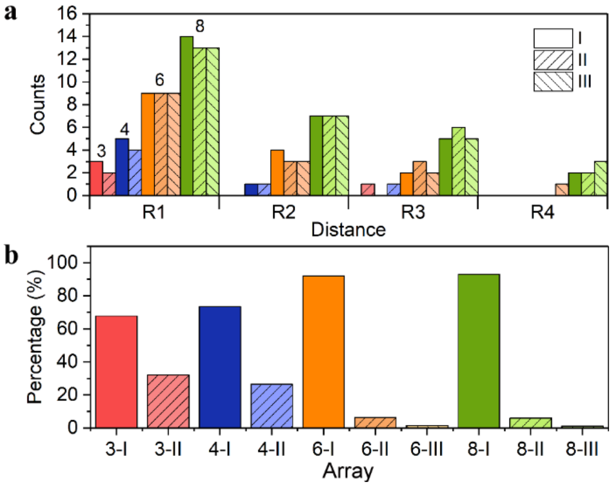

Supplementary Figure 3. Analysis of optical matter isomers. **a,** Percentage of different isomers observed in the experiments. **b**, Counts of NP pairs with pairwise distances equal to R1-R4 in different isomers with 3, 4, 6 and 8 NPs. R1 to R4 denote the first to fourth nearest neighbor distances from the first particle.

Supplementary Table 1. Statistics of optical matter isomers. Counts of NP pairs with pairwise distances equal to R1-R6 (the first to sixth nearest neighbor distances from the first particle) for optical matter isomers with 3-9 NPs, and counts of frames and percentages of the corresponding isomers observed in experiments.

Array	Interparticle Distance						Number	Percentage
	R	1.732R	2R	2.646R	3R	3.456R	of frames	(%)
**3I**	3						1676	67.91
**3II**	2		1				792	32.09
**4I**	5	1					13401	73.53
**4II**	4	1	1				4824	26.47
**5I**	7	2	1				5812	79.88
**5II**	6	3	1				1464	20.12
**6I**	9	4	2				6545	91.96
**6II**	9	3	3				462	6.49
**6III**	9	3	2	1			110	1.55
**7I**	12	6	3				11914	91.49
**7II**	11	5	4	1			1013	7.78
**7III**	11	5	3	2			82	0.63
**7IV**	11	4	3	2	1		13	0.10
**8I**	14	7	5	2			21308	92.63
**8II**	13	7	6	2			1364	5.93
**8III**	13	7	5	3			245	1.07
**8IV**	13	7	5	2	1		65	0.28
**8V**	13	6	4	4	1		21	0.09
**9I**	16	9	7	4			16467	76.62
**9II**	16	8	7	4	1		3008	14
**9III**	16	9	6	4	1		1547	7.2
**9IV**	15	9	7	4	1		368	1.71
**9V**	16	8	7	4		1	101	0.47

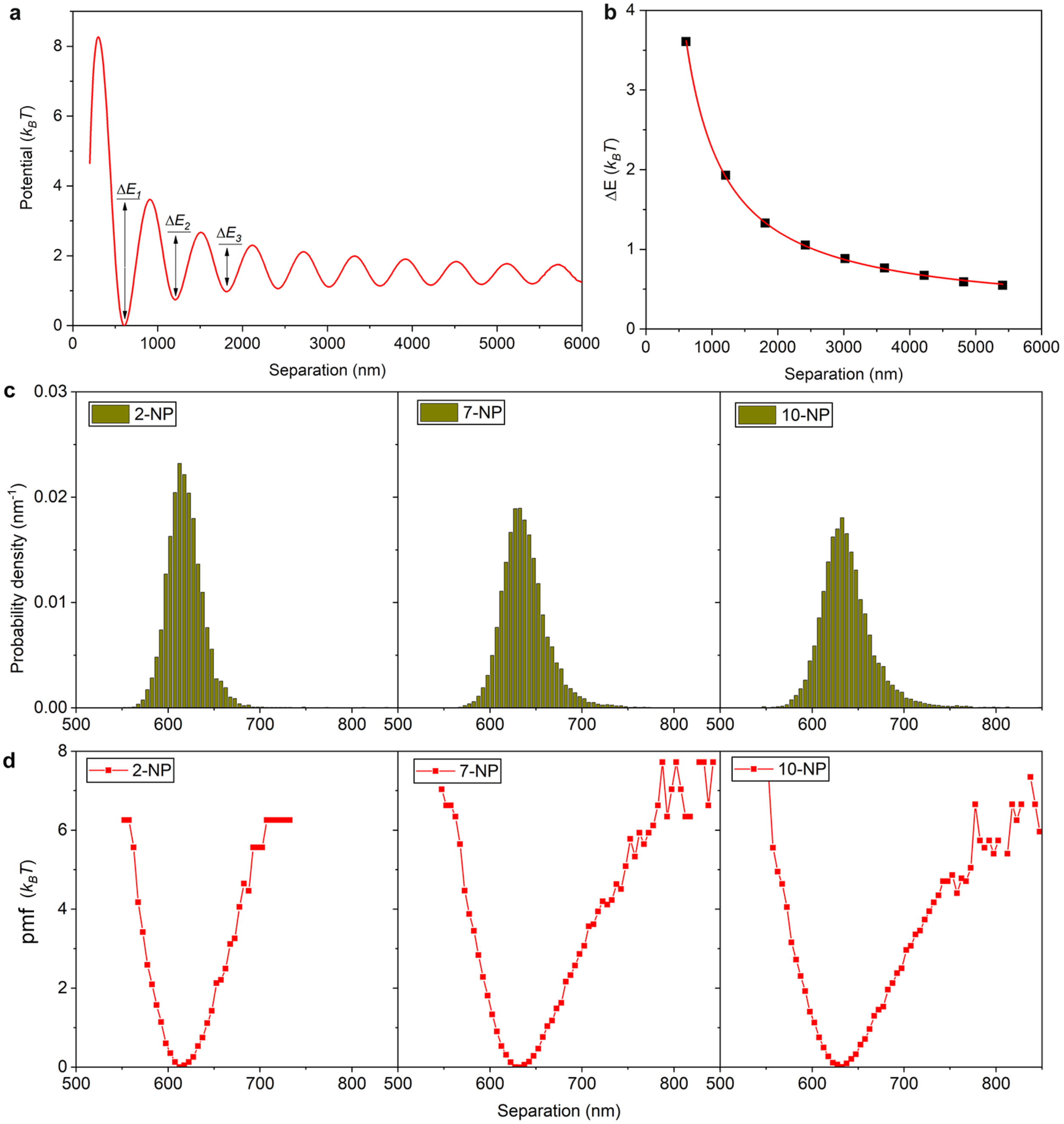

Supplementary Figure 4. Optical binding strength of optical matter arrays formed in circularly polarized light. **a,** Theoretical effective optical binding potentials between two NPs at different interparticle separations. **b,** Data points are the potential well depths (ΔE as defined in panel) at different optical binding separations, d. The red fit curve follows a function of ΔE=0.18+2099.95/d. **c**, The probability densities of separation distribution between first nearest neighbors in 2-NP, 7-NP and 10-NP arrays observed in experiments. **d**, The corresponding potentials of mean force (pmf) of optical binding in these arrays.

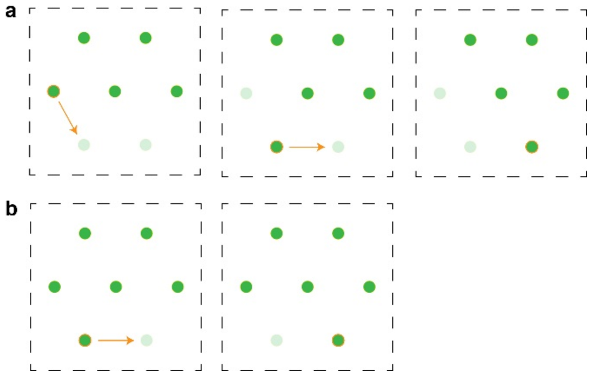

Supplementary Figure 5. Structure reconfiguration of arrays **a**, 5-I and **b**, 6-I created by relocating 1 NP.

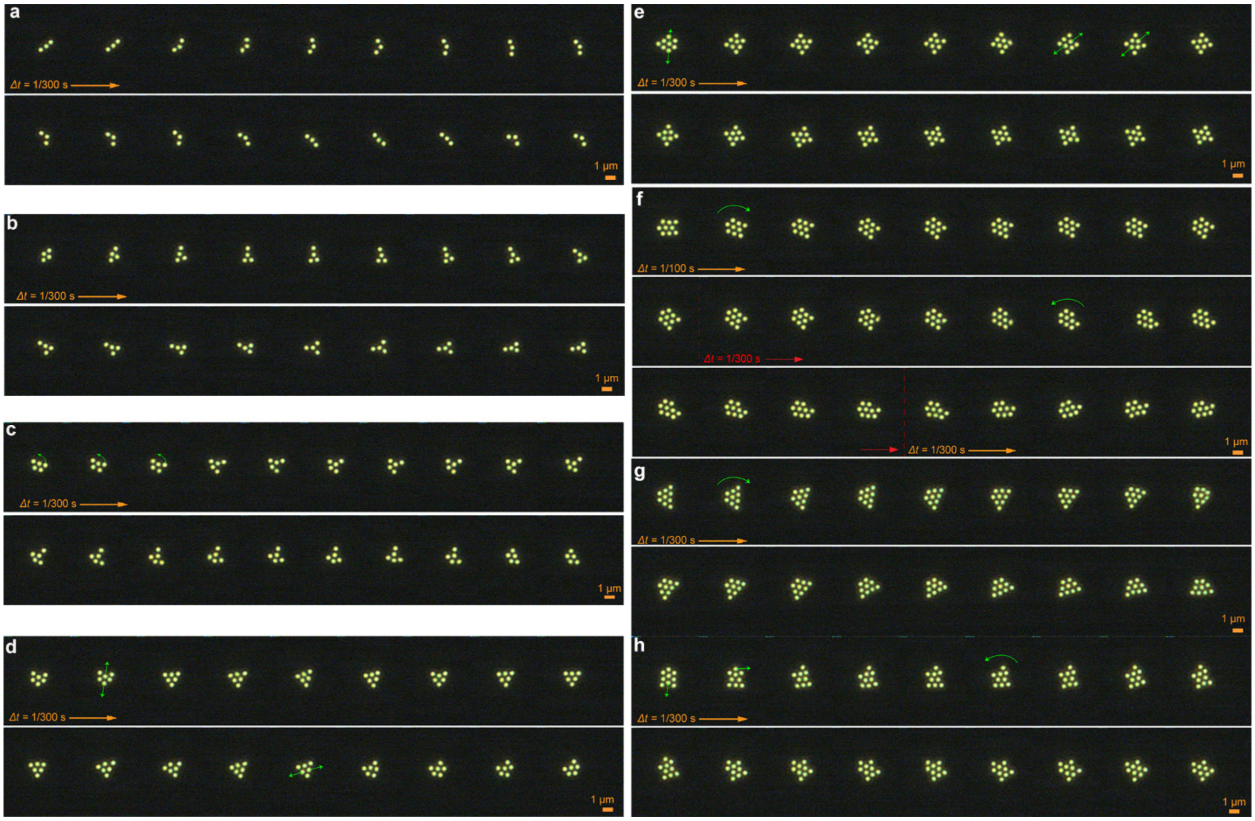

Supplementary Figure 6. Dark-field optical images of optical matter arrays with structural transition and rotation. **a**, 3-NP arrays; **b**, 4-NP arrays; **c**, 5-NP arrays; **d**, 6-NP arrays; **e**, 8-NP arrays; **f-h**, 9-NP arrays.

Supplementary Note 2. Influence of the spin of single NPs to the rotation of optical matter arrays

The spin angular momentum of the incident photons couples into the spinning of the individual NPs (primarily through absorption). Here we consider the influence of the spin of single NPs to the rotation of the entire NP arrays with Stokes drag. Supplementary Figure 7 shows that the electrodynamic torque associated with particle spin is generally positive, even when the orbital torque on the cluster is negative. The spin torque is about 50 times weaker than the orbital torque. The spinning NPs in the fluid cause fluid flow and possible hydrodynamic coupling in addition to the electrodynamic coupling. However, the hydrodynamics cannot cause the observed negative orbital torque since the NP spinning exhibits a positive torque and will therefore lead to a positive contribution to the orbital motion through hydrodynamic coupling. Furthermore, there is no retardation length scale for the hydrodynamic interactions in this system, so the orbital torque cannot switch signs like it does in the electrodynamic coupling case. Thus, the experimentally observed negative orbital torque occurs despite the presence of hydrodynamic interactions that may in some cases be working against the electrodynamic torque.

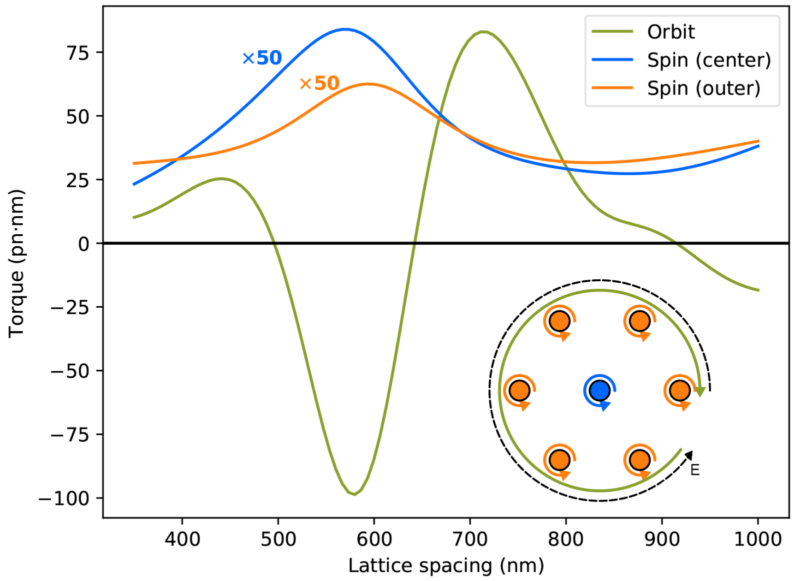

Supplementary Figure 7. Spin and orbital torque as a function of the lattice spacing in a 7-NP array. The dashed arrow in the inset indicates the rotation direction of the electric vector of light, and the solid arrows indicate the rotation directions of individual NPs or the whole array.

Supplementary Note 3. Influence of interparticle separation on optical torque

Here we check whether the interparticle separations of optical matter arrays plays a role in their rotational dynamics and potentially the sign of optical torque. There are several possible ways to tune the interparticle separations, such as adding ionic compounds or surfactants into the solution to change the electrostatic screening and electrical double layers surrounding the NPs, or controllably adding compressive optical forces with intensity or phase gradients. Here we adjust the interparticle spacing by adding cetyl trimethylammonium bromide (CTAB) into the Ag NP solution. The positive charge of the CTAB molecule changes the electrostatic interactions between the otherwise nominally negative charged PVP-coated Ag NPs. Therefore, the net reduction of the negative charge on the Ag NPs causes a reduction of their separations. Supplementary Figure 8 shows an example where the crossover from positive to negative optical torque can happen between 2-NP and 3-NP arrays when the interparticle separations are smaller.

Supplementary Figure 9 shows another example where both the interparticle separation and the configuration can determine the rotation direction and thus the sign of optical torque in a 7-NP array (also see Movie S3). This array shows three different rotation behaviors (Supplementary Figure 9a). In time region I, it is a 7-II optical matter array but with smaller interparticle separation compared to the NP sample without CTAB molecules (Supplementary Figure 9b). The smaller separation changes the rotation direction of the 7-II optical matter array from counterclockwise (i.e., positive optical torque as shown in Supplementary Figure 9c) to clockwise (i.e., negative optical torque in Supplementary Figure 9d). This agrees with the prediction from the simulations (see Supplementary Figure 16 where the 7-II in panel a has smaller interparticle separations and shows clockwise rotation, and 7-II in panel b has larger separations and shows counterclockwise rotation). In time region II, the array configuration is unstable due to fluctuations of the NPs, and no persistent rotation is observed. In time region III, two NPs adopt an even smaller separation, switching from ~600 nm to ~400 nm as shown in Supplementary Figure 9e (perhaps due to inhomogeneous adhesion of CTAB molecules to the NPs), and the array shows a reversal of the rotation direction (i.e., from negative to positive optical torque). These two examples clearly demonstrate that the interparticle separation and the structure of the array strongly affects the (sign of) optical torque.

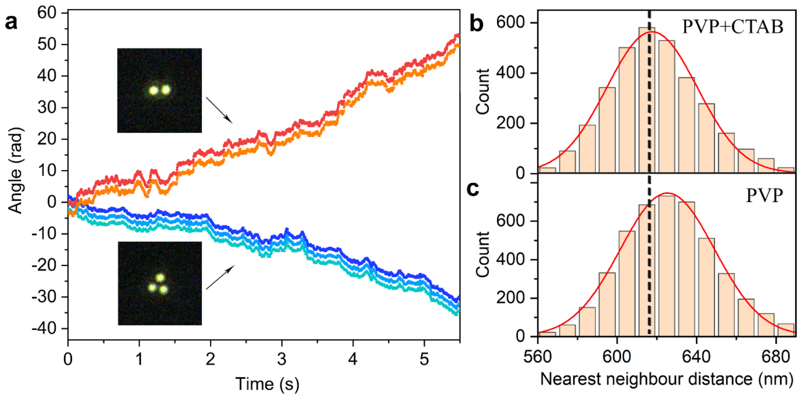

Supplementary Figure 8. Crossover from positive to negative optical torque occurs between 2-NP and 3-NP arrays when the interparticle separations are smaller. **a**, Time trajectories of experimentally measured orientations of individual NPs in a dimer and a trimer relative to their centers of mass when CTAB is added to the Ag NP solution. **b**. Histograms of interparticle

separations for the 3-NP array. **c**, Histograms of interparticle separations for a 3-NP array with only PVP-coated Ag NPs for comparison.

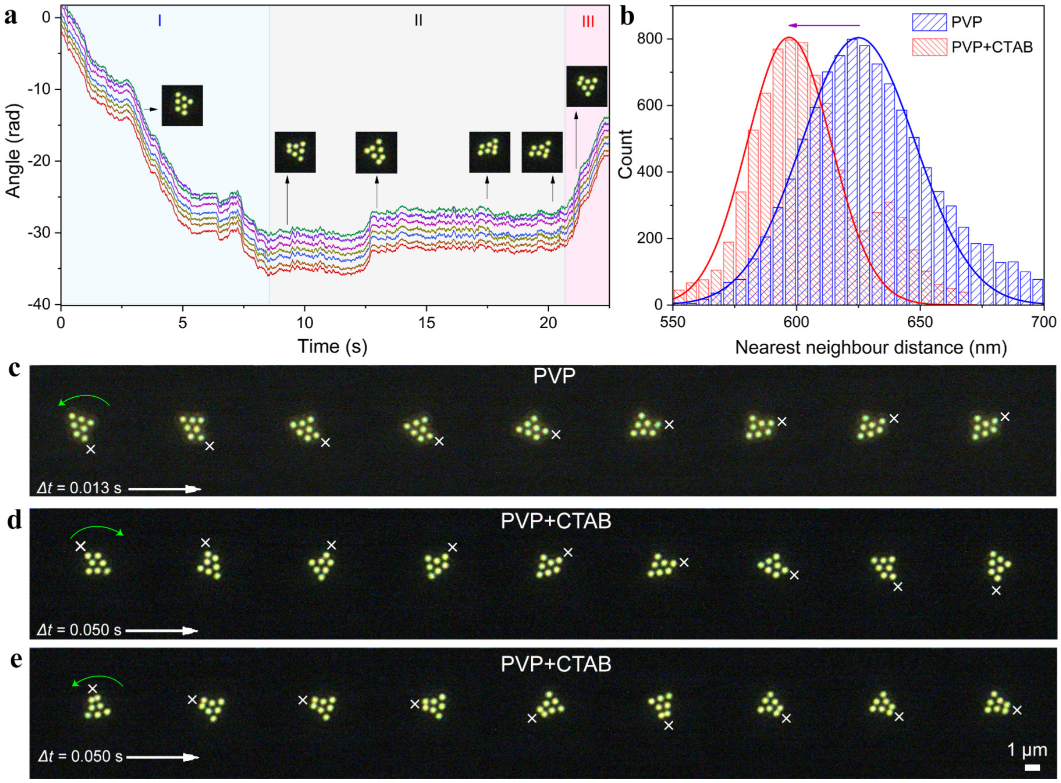

Supplementary Figure 9. Optical torque reversal in optical matter arrays with different interparticle separations. **a**, Time trajectories of orientations of individual NPs in a 7-NP array relative to its center of mass when CTAB molecules are added into the Ag NP solution. Three types of dynamics are observed as illustrated in the three time regions: time region I shows clockwise rotation (i.e., negative torque), time region II are nearly stationary due to fluctuation of the NP configuration, and time region III shows counterclockwise rotation (i.e., positive torque). Representative optical images at different times are shown in the insets. **b**, Histograms of interparticle separations for the array in the region I (labeled as PVP+CTAB). The separations for a similar 7-NP array (as shown in panel c) with only PVP-coated Ag NPs is also shown for comparison (labeled as PVP). **c**, Optical images of a 7-NP array with only a PVP coating. **d**, Optical images of the 7-NP array in time region I of the PVP+CTAB sample. **e,** Optical images of the 7-NP array in the time region III of the PVP+CTAB sample, where two NPs have a small separation (~400 nm), leading to reversal of the rotation direction.

Supplementary Note 4. Influence of geometry on optical torque

Different structures (configurations) of NPs in the arrays result in different (and even opposite) optical torques at a certain lattice constant as shown in Supplementary Figure 10a,b. The optical torque curves look similar, but the different values of interparticle separation giving zero-torque indicate that different structures (isomers) could exhibit different torque crossover behavior. The optical torques are opposite for arrays 3-I and 3-II at a lattice constant of 575 nm, and the 9-III curve has a shoulder with positive torque for lattice constant values between 400 and 500 nm, while the curves for 9-I and 9-II curves have negative torque throughout that range.

Examining Supplementary Figure 2b, one notices that the positions 11 and 12 are actually exchangeable for both arrays 11-I and 12-I. Similarly, positions 9 and 10 are the same for 10-I. A spiral is formed if the 9th particle in 9-I took the position 10 (i.e., forming 9-III), and the one connected negative torque region at the 9-NP-array shown in Fig. 3c in the main text then separates into two. This illustrates a clear trend for the torque evolution from the 8-NP-array to larger arrays. In addition, the secondary negative torque region at small lattice constants appears from the 8-NP-array, which is coincident with the start of the 2nd hexagonal layer in a lattice (see Supplementary Figure 10c). To examine the possible correlation between the number of negative torque regions and number of layers, we extended our calculation to the 3rd layer, but no additional negative torque regions appear for 20 or more NPs (Supplementary Figure 10d).

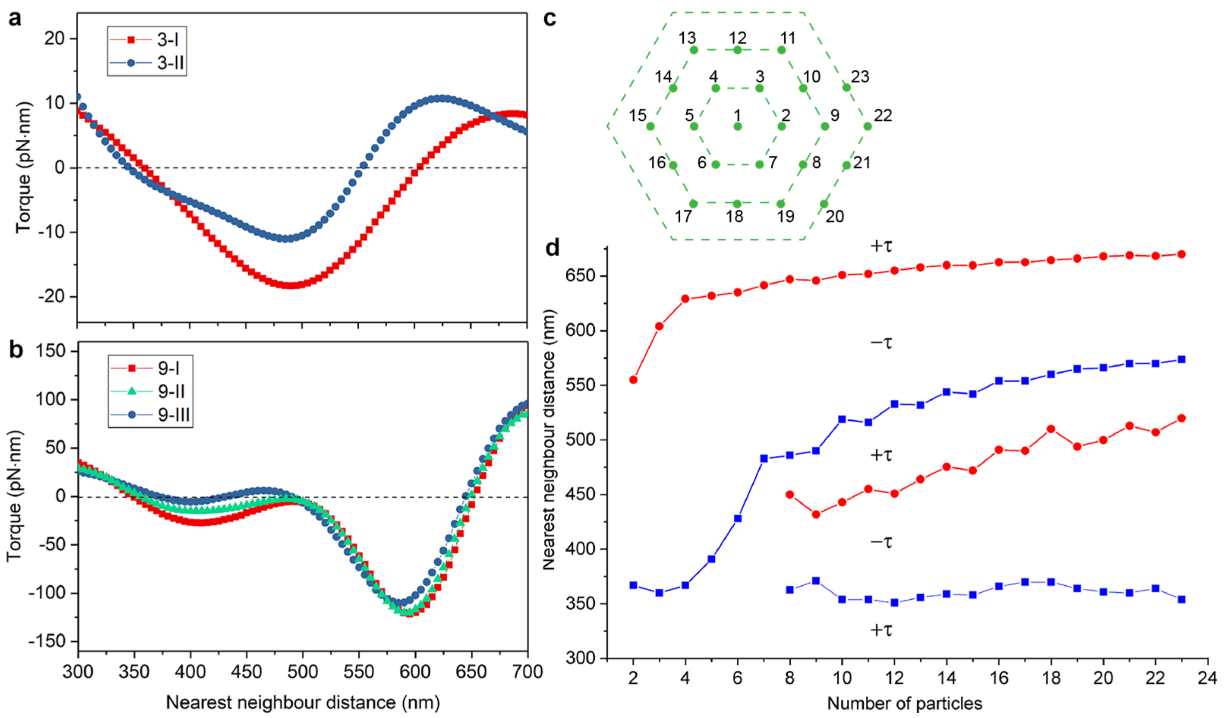

Supplementary Figure 10. Calculated optical torque of hexagonal lattice optical matter arrays. **a,** Optical torques in two configurations for 3-NP-arrays and **b,** three configurations for 9-NP-arrays with increasing lattice constants. Type I, II, and III structures follow the configurations shown in Supplementary Figure 2. **c,** Illustration of optical matter arrays where up to 23 NPs are added in a sequential manner. **d,** The corresponding optical torque diagram where the solid symbols define boundaries of torque reversals.

Supplementary Note 5. Influence of the coverslip near the particles

Our simulations assume the NPs are surrounded by a homogenous environment (i.e., water), but in the experiments, the Ag NPs are pushed by optical scattering forces against the upper coverslip surface. We consider whether the proximity to the glass coverslip (and the glass-water interface) impact the interactions among the NPs. As shown in Supplementary Figure 11a, the glass surface reflects a small portion of the incident light causing interference in the axial direction. However, it is of very small amplitude compared to a standing wave created by reflection from a gold mirror. Nevertheless, we checked the influence of the glass surface on the optical interaction with the NPs. Since both the NP and glass surfaces are negatively charged in our experiments, electrostatic repulsive forces keep them separated.

We first estimate the separation d between the Ag NPs and glass surface as illustrated in the inset of Supplementary Figure 11b. The total potential energy $W(d)$ of a Ag NP near a glass surface is the sum of van der Waals potential energy $W_A(d)$, the potential energy of electrostatic double layer repulsion $W_R(d)$, the gravitational potential energy $W_g(d)$, and the potential energy from the laser scattering $W_{scat}(d)$. These potentials are given by[1]

$$W(d) = W_A(d) + W_R(d) + W_g(d) + W_{scat}(d), \tag{11}$$

$$W_A(d) = -\frac{A}{6}\left(\frac{r}{d} + \frac{r}{2r+d} + \ln\frac{d}{2r+d}\right), \tag{12}$$

$$W_R(d) = 16\varepsilon r\left(\frac{k_B T}{z_p \mathrm{e}}\right)^2 \tanh\left(\frac{z_p \mathrm{e}\psi_p}{4k_B T}\right)\tanh\left(\frac{z_p \mathrm{e}\psi_s}{4k_B T}\right)\exp(-\kappa d), \tag{13}$$

$$W_g(d) = -\frac{4}{3}\pi r^3(\rho_{Ag} - \rho_{H_2O})gd, \tag{14}$$

$$W_{scat}(d) = F_{scat} d, \tag{15}$$

where A is the Hamaker constant, r is the NP radius, ε is the solvent permittivity, k_B is Boltzmann's constant, T is the absolute temperature, z_p is the particle valence, e is the elemental charge, ψ_p and ψ_s are the NP and glass surface Stern potentials, κ is the inverse Debye length, ρ is density, g is gravitational acceleration, and F_{scat} is the scattering force. The parameter values we used are: A= 3×10^{-20} J,[2] $\kappa = 0.0445$ nm^{-1},[1] $\psi_p = -56$ mV, $\psi_s = -100$ mV, $z_p = 1$, $T = 300$ K, $\rho_{Ag} = 10.5$ g cm^{-3}, $\rho_{H2O} = 1$ gcm^{-3}, g = 9.8 m s^{-2}, and $F_{scat} = 9.32\times10^{-13}$ N (calculated by FDTD simulation).The calculated total potential energy is plotted in Supplementary Figure 11b.The equilibrium position of the Ag NP near the glass surface is determined by the local minimum of the potential energy, which is at $d = 50$ nm surface-to-surface. The potential well allows estimating the z-axis thermal fluctuation of a NP with thermal energy of $0.5k_BT$; the range is 18 nm as defined by the intersection points of the dashed line with the potential curve. This is much smaller than the particle size (150 nm dia.), so we assume the particles are located in one plane.

We built simulation models with a glass surface near Ag NPs at $d = 50$ nm, and recalculated the optical torque "phase diagram" near the optical binding separation of 600 nm. The results are shown in Supplementary Figure 11c, where the torque crossover for Ag nanoparticles without a glass surface is also shown for comparison. They both show the same feature of torque crossover depending on the interparticle separation and particle number, and the reversal separations that are reasonably close (with a maximum difference of 9 nm) for the 2-13 NP arrays. In addition, we calculated the electric field distributions of two Ag NPs at the optical binding separation with and

without a glass surface (Supplementary Figure 11d,e). The results are nearly the same as without the glass. Therefore, the simulations without a glass surface manifest all the important features of optical forces and torques, including the torque crossover observed in our experiments.

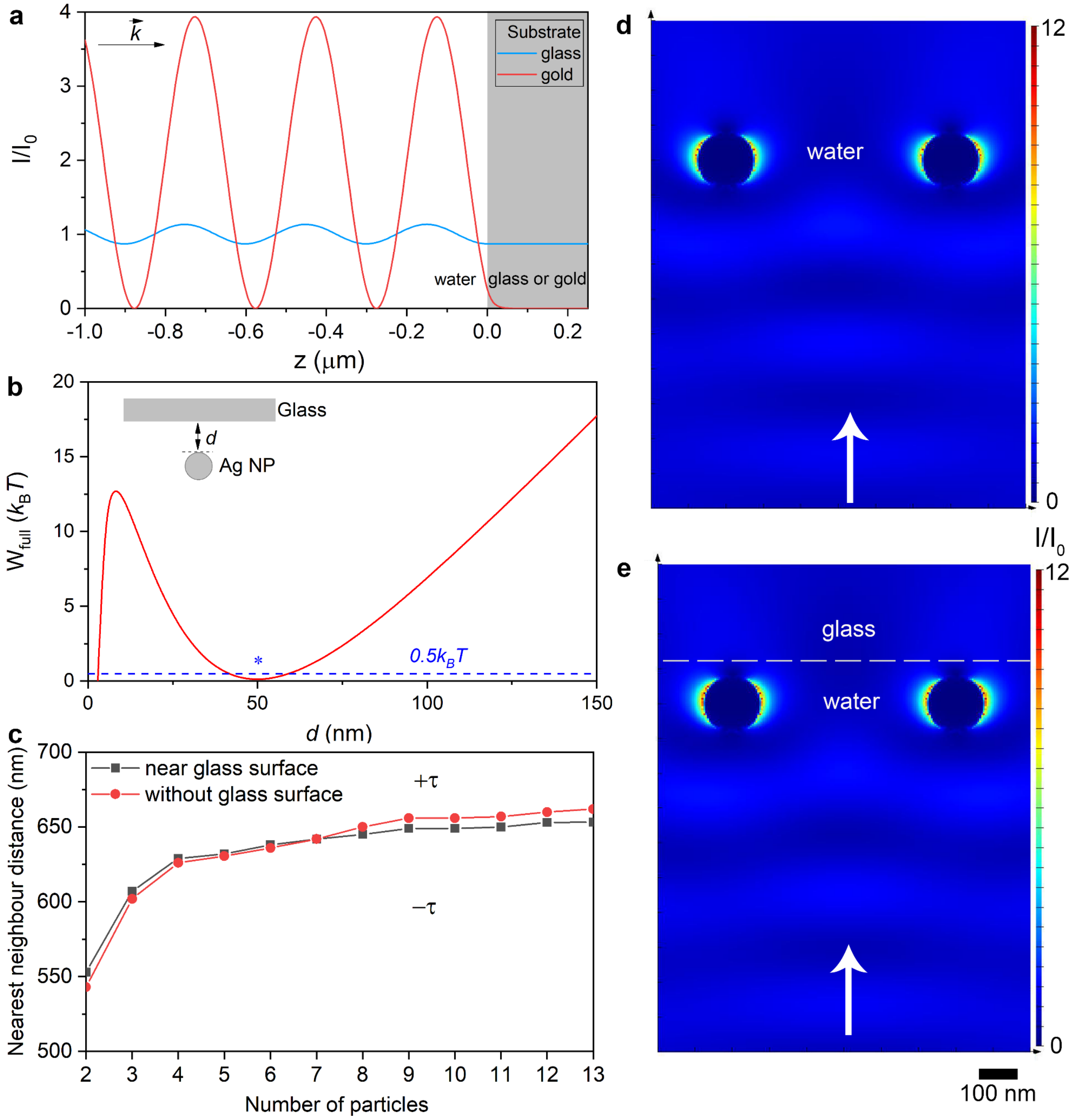

Supplementary Figure 11. Influence of a coverslip surface near the Ag NPs. **a**, Calculated intensity variation (blue color) for propagation of 800 nm wavelength light from water to a glass substrate (refractive index n = 1.517). The result for a gold substrate is also shown (red color) for comparison. **b**, The calculated DLVO potential energy of a Ag NP over a coverslip surface. The dashed line is the thermal energy that each NP has in the z dimension. The width of the potential well allows thermal fluctuations in the axial direction in the range of $d = 42$ to 60 nm. **c**, A portion of the optical torque "phase diagram" for Ag NP optical matter arrays with and without a glass surface. The separation of the Ag NP and glass surface is 50 nm edge-to-edge. **d**, Calculated intensity distribution (normalized to the intensity of incident light) for two Ag NPs illuminated by 800 nm light in water. **e**, Calculated intensity distribution near a glass surface.

Supplementary Note 6. Photothermal effect and thermophoretic force

The laser used to assemble the Ag NPs heats them which creates thermal gradients in the surrounding water medium and thermophoretic forces. The heat source density inside a Ag NP is given by[3]

$$q(r) = \frac{\omega}{2}\mathrm{Im}[\varepsilon(\omega)]|\boldsymbol{E}(r)|^2 \tag{16}$$

where $\boldsymbol{E}(r)$ is the complex amplitude of the electric field, ε is the permittivity of silver and ω is the frequency of light. The function q(r) represents the energy source coming from electromagnetic losses in the Ag NPs. The temperature can be calculated using the heat diffusion equation:

$$\rho C_\mathrm{p}\nabla \cdot (T\bar{U}) = \kappa\nabla^2 T + q(r), \tag{17}$$

where $\bar{U}$ is the velocity field, ρ, C_p, T and κ are the density, heat capacity, temperature, and thermal conductivity of the material, respectively. The convective term $\nabla \cdot (T\bar{U})$ can be ignored for heat transfer at nanometer scale.[4] Thermophoretic force can then be calculated from the temperature gradient using

$$F_\mathrm{tph} = -6\pi\mu R D_\mathrm{T}\nabla T, \tag{18}$$

where μ is the viscosity of water and D_T is the thermodiffusion coefficient. Most of the measured coefficients have values in the range of 10^{-12} to 10^{-11} $m^2\ s^{-1}\ K^{-1}$, according to Piazza's review[5].

Our numerical study of the photothermal effect is performed using COMSOL Multiphysics software. The parameters used are: laser intensity I = 4 mW μm^{-2}, wavelength λ = 800 nm, thermal conductivity of water k_water = 0.6 W s^{-1} K^{-1}, thermal conductivity of glass k_glass = 1.4 W s^{-1} K^{-1} and $D_\mathrm{T} = 1\times10^{-11}$ $m^2\ s^{-1}\ K^{-1}$. The results for a Ag NP in bulk water and near a glass/water interface are shown in Supplementary Figure 12. Both show mild photothermal effects: the maximum thermophoretic force is ~1 pN for a calculated maximum temperature increase of about 7 K. The thermophoretic force decays rapidly with increasing interparticle separation as shown in Supplementary Figure 13, and therefore the optical binding force dominates for interparticle separations larger than 300 nm. The profiles of optical binding forces between two NPs with and without thermophoretic force are similar, but the thermophoretic force will increase the equilibrium separation from 600 nm to 612 nm.

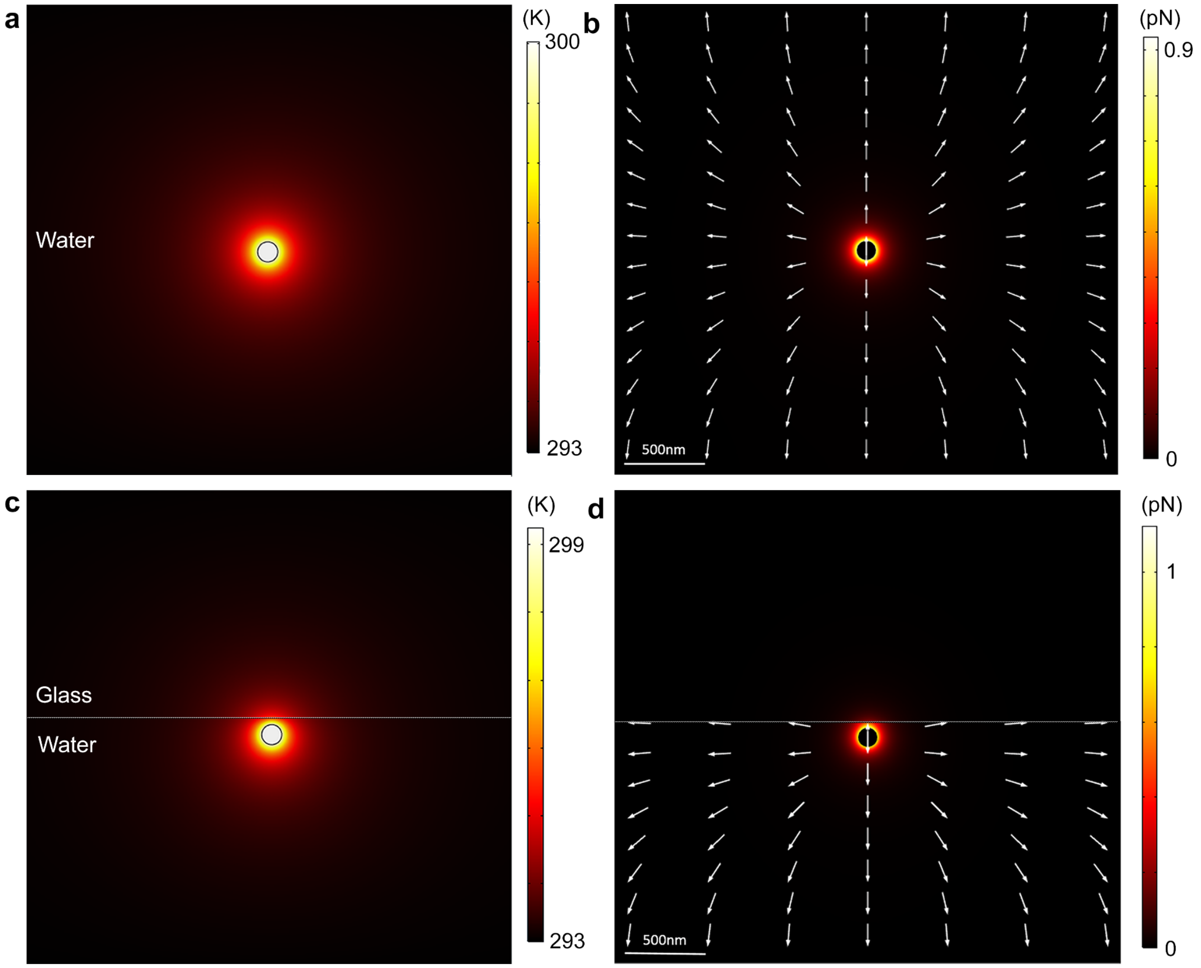

Supplementary Figure 12. Photothermal effect of a laser illuminated Ag NP (150 nm dia.). **a**, The calculated temperature profile of a Ag NP in water. **b**, The distribution of thermophoretic force of a Ag NP (shown as the black sphere at a fixed position) to another one (not shown on the image but it maps the thermophoretic forces in the water space) due to the photothermal effect. The arrows indicate the force directions. **c**, The calculated temperature profile of a Ag NP near the glass surface. **d**, The distribution of thermophoretic force on another Ag NP near glass surface. The arrows indicate force directions while the colors give the magnitudes of the thermophoretic forces. The separation of the Ag NP and glass surface is 50 nm.

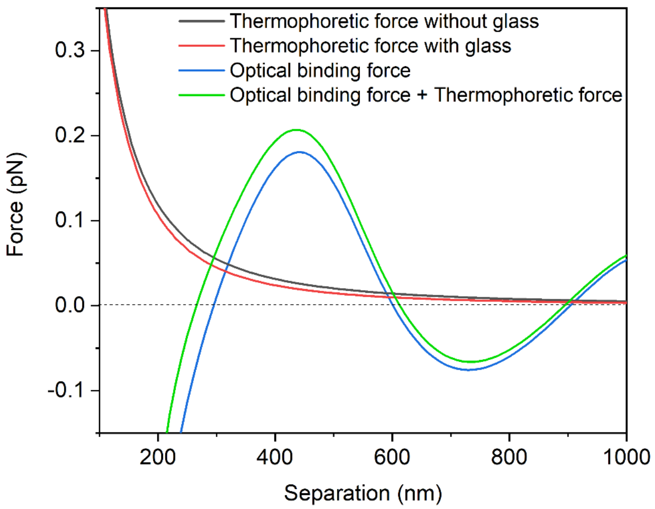

Supplementary Figure 13. The calculated thermophoretic force between two Ag NPs in the horizontal direction of Supplementary Figure 12b and d (i.e., perpendicular to the laser propagation direction) with and without a glass surface. The optical binding forces between two NPs obtained from FDTD simulation and the sum of the optical binding force and the thermophoretic forces (without glass) are also plotted for comparison. The optical binding forces are calculated with the same laser intensity as that used for calculating the thermophoretic forces.

Supplementary Note 7. FDTD-particle dynamics simulations of optical matter isomers

Our FDTD-particle dynamics method can capture both the light-driven self-organization and orbital motion of optical matter isomers in circularly polarized beams of light. Supplementary Figure 14 shows an example where 9 Ag NPs (150 nm dia.) randomly distributed in water self-organize into a 9-I or a 9-III array upon illumination by a λ = 800 nm LHCP plane wave. Supplementary Figure 15 further shows that the angular velocities of the optical matter arrays calculated by the simulations agree well with the experimental observations.

The simulated trajectories of various optical matter isomers with light-induced rotation are shown in Supplementary Figure 16, where two conditions for nanoparticles without and with surface charges have been considered. The rotation behaviors are typically different for optical matter arrays with different numbers of particles, and they can even be different for optical matter isomers with the same number of particles. For example, the 9-I and 9-II arrays rotate clockwise while 9-III rotates counterclockwise, the same as that observed in experiments (Supplementary Figure 17). Predicted deviations of some optical matter arrays from hexagonal lattices are also seen in experiments, for example, the "curved" 3-II (Supplementary Figure 6a) and the Y-shaped 4-II (Supplementary Figure 6b). The calculated optical torques in optical matter isomers (Supplementary Figure 18) show that the optical torques generally decrease and thus change from positive to negative when the arrays involve more particles. The configurations of the particles also strongly influence the magnitude and sign of optical torque. The optical torque reversal or cross-over shifts to larger particle numbers in the arrays when repulsive electrostatic interactions are included and the interparticle separation increases.

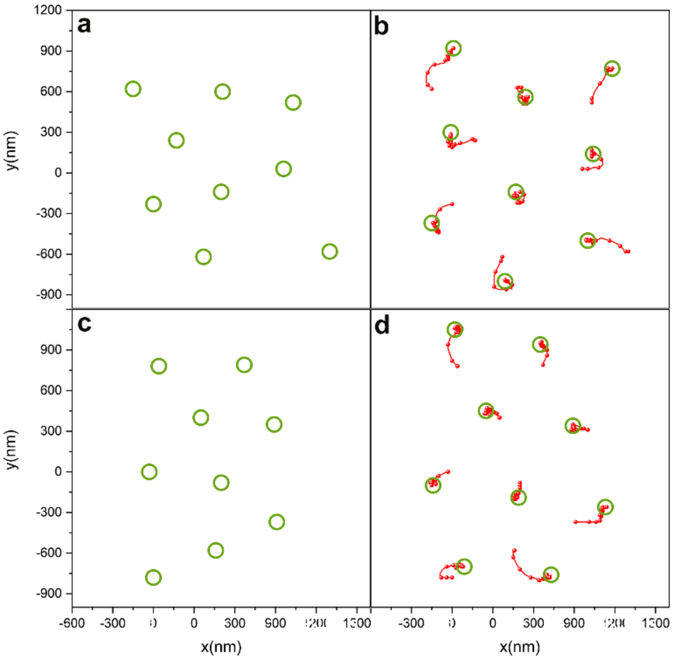

Supplementary Figure 14. Self-organization and orbital motions of 9 NPs in a LHCP laser beam computed by a FDTD-particle dynamics simulation approach. **a & c,** The random initial positions of the NPs. **b & d,** The corresponding trajectories of the NPs self-organized into two different arrays. The open circles show the final positions of the NPs. A constant surface charge of -1.33×10^{-18} C is assumed on each particle surface.

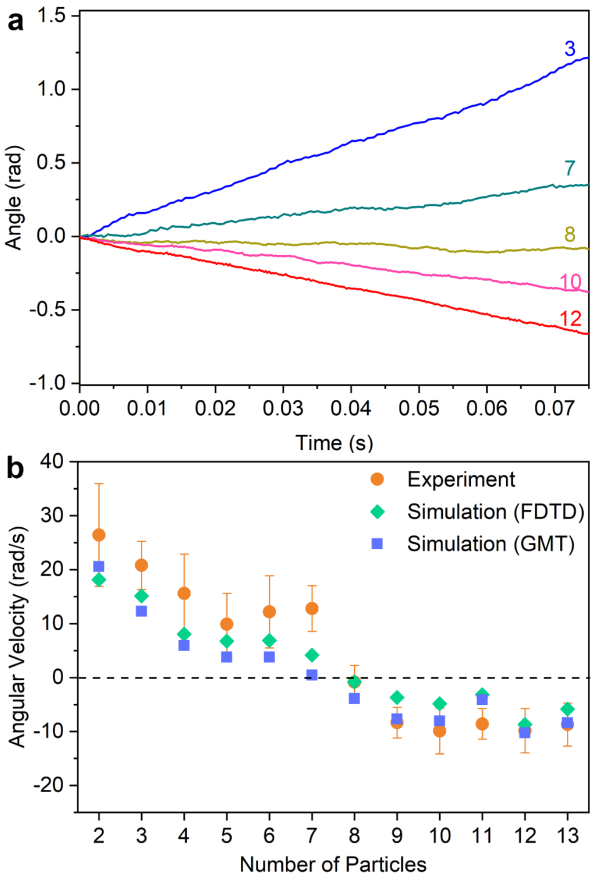

Supplementary Figure 15. Simulated orbital motion of type-I optical matter arrays relative to their centers of mass. **a,** Time trajectories of the orientations of several optical matter arrays simulated by the FDTD-particle dynamics approach. The numbers indicate the number of Ag NPs in the array. **b,** Average angular velocity of optical matter arrays as a function of the number of particles. Comparison of the results from the experiments, the FDTD-particle dynamics and GMT-Langevin dynamics simulations. All results are nearly in quantitative agreement when the interparticle separations in the simulations match with the experimental values.

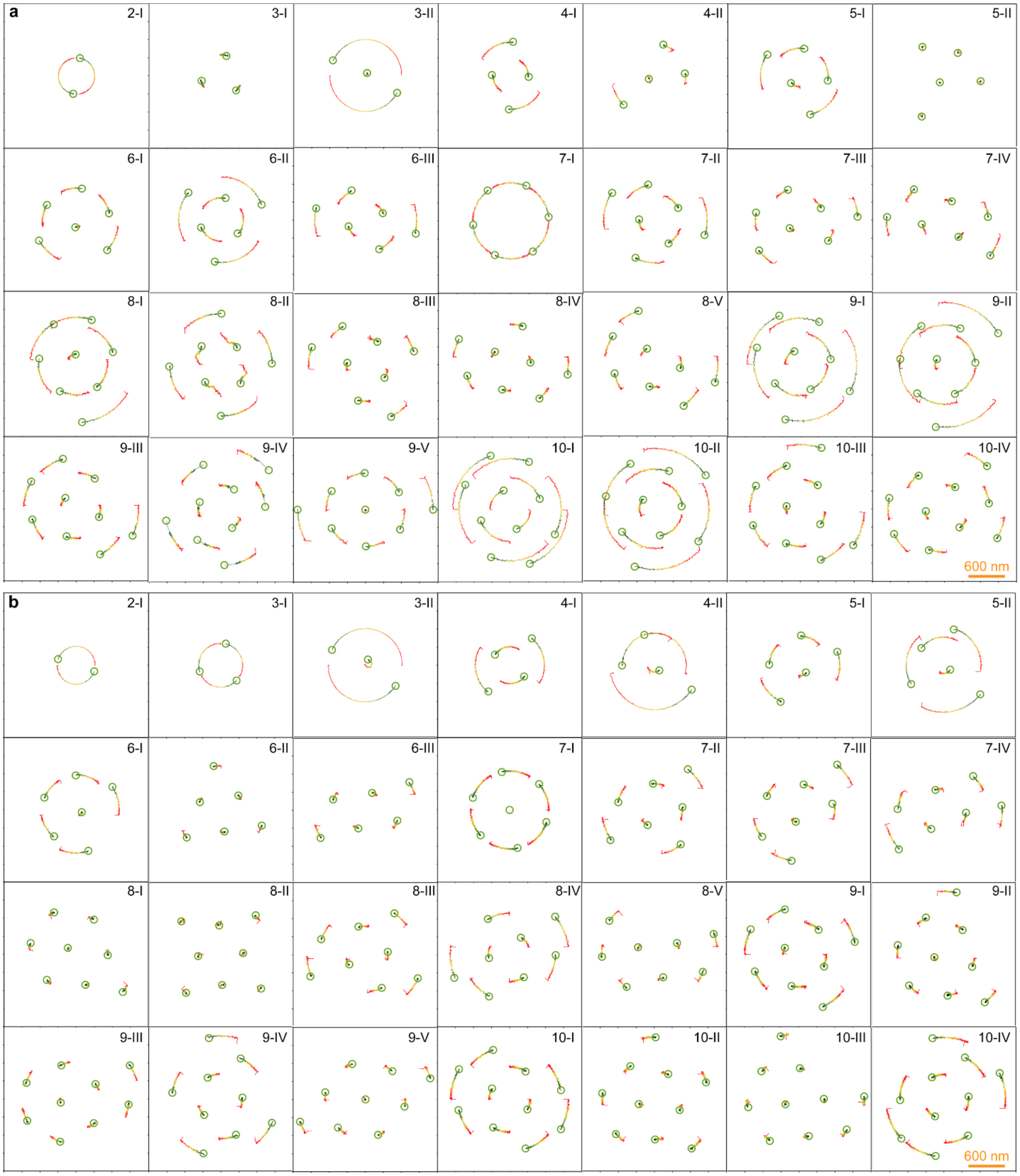

Supplementary Figure 16. Simulated trajectories of various optical matter isomers exhibiting light-induced rotation. **a**, Particle arrays with pure optical binding interactions. **b**, Particles have both optical binding and electrostatic interactions. The electrostatic interactions in the simulation we obtained when using a constant surface charge of -1.33×10^{-18} C on each particle surface, the same as that used to calculate other optical matter arrays in Fig. 4b in the main text. All trajectories proceed from red to green in time and end at the green circles.

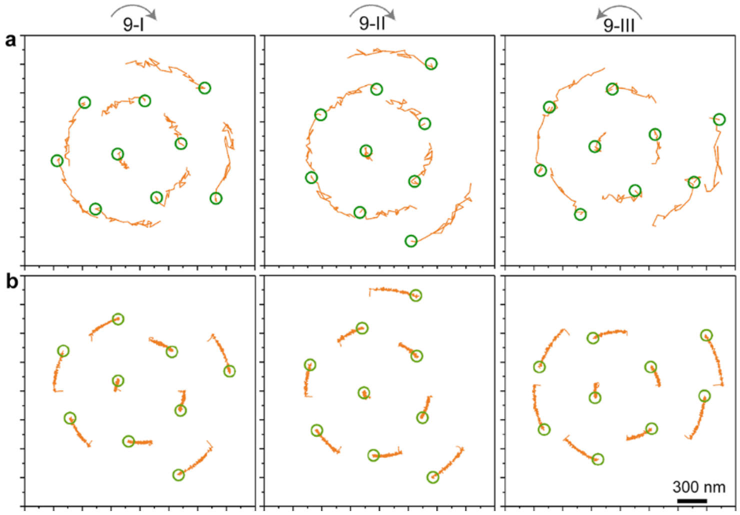

Supplementary Figure 17. Trajectories of three 9-NP-arrays relative to the center of mass of each array. **a**, Trajectories of arrays observed in experiments. **b**, Trajectories from simulations. The green open circles indicate the final position of each particle. The simulation results agree well with the experimental results.

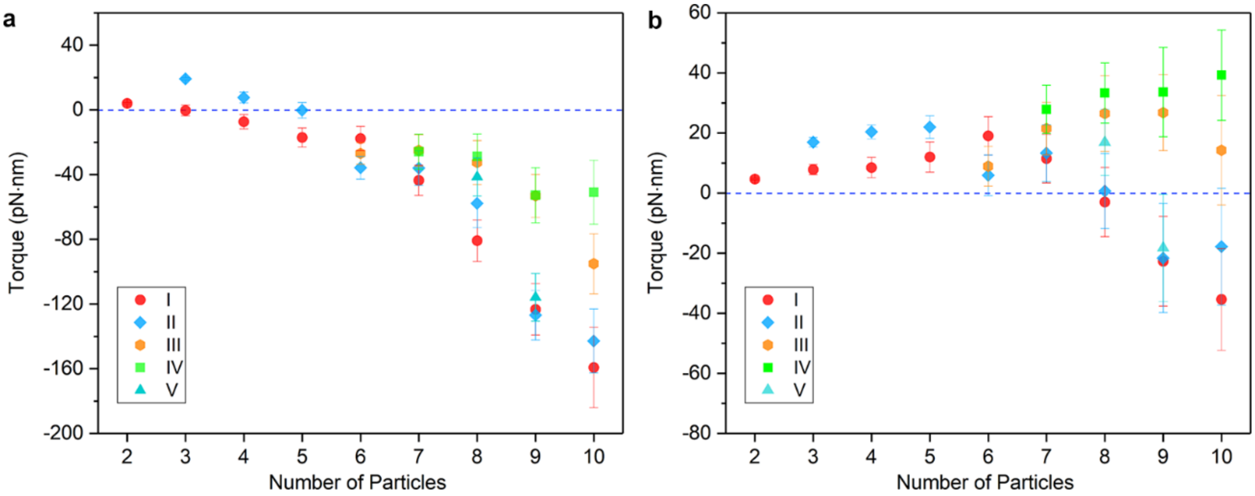

Supplementary Figure 18. Calculated optical torques in optical matter isomers. **a**, Particle arrays with pure optical binding interactions. **b**, Particle arrays with both optical binding and electrostatic interactions. The colors indicate different configurations of the isomers.

Supplementary Note 8. Influence of laser wavelength on optical torque

An additional way to control the interparticle separation of optical matter arrays is by tuning the laser wavelength, which determines the optical binding separation. However, the electrodynamic interactions will also change with wavelength. Supplementary Figure 19a shows trajectories from FDTD-particle dynamics simulations for type-I optical matter arrays with 2 to 11 NPs, where all the arrays show counterclockwise rotation (i.e., positive optical torques). The 710 nm illumination generates a different sign of optical torque to the 9-I, 10-I and 11-I arrays compared to the results obtained at 800 nm (Supplementary Figure 16b), clearly. We performed experiments by tuning the laser wavelength to 710 nm and our observations agree with the simulations. The 9-I, 10-I and 11-I arrays all rotate counterclockwise (Supplementary Figure 19b) with positive optical torques. The interparticle separations for the arrays in both simulation and experiment are around 600 nm (Supplementary Figure 19c,d), smaller than the separations (~640 nm) of arrays assembled by 800 nm laser illumination. These results demonstrate that the laser wavelength also plays an important role in determining the (sign of) optical torque.

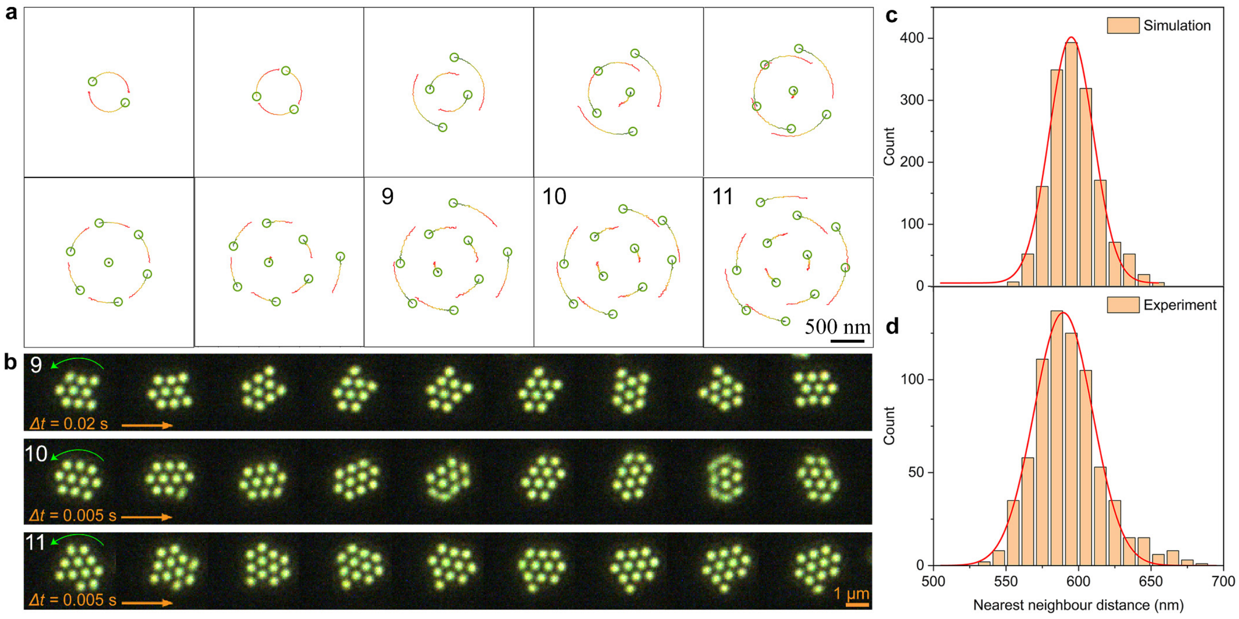

Supplementary Figure 19. Rotational dynamics of optical matter array assembled by a LHCP laser beam with $\lambda = 710$ nm wavelength. **a**, Trajectories from FDTD/particle dynamics simulations for the type-I optical matter arrays. **b**, Optical images of 9-I, 10-I and 11-I arrays that all rotate in the counterclockwise direction, the same direction as predicted by simulation. **c** & **d**, Histograms of interparticle separations for 9-I arrays in simulation and experiment.

Supplementary Note 9. Simulations with irregular NPs

A well-known issue for experiments with metal NPs is that they are usually not ideal spheres. This can be seen in Supplementary Figure 20a where the Ag NPs used in our experiments have facets and irregular surfaces. To test whether irregular NPs could still show light-driven self-organization and orbital motions in circularly polarized light, we performed simulations using particles with rough surfaces (Supplementary Figure 20b). Here each particle is composed of many thin polygonal slices, each slice has a random shape but has also some correlation with the adjoining slices. Therefore, the synthetic particle still has an overall spherical shape but with facets and rough surfaces, and every particle is unique, similar to the real Ag NPs used in the experiments. Simulation results for irregular NPs without surface charges are shown in Supplementary Figure 20c,d. The results show that light-driven self-organization and rotation can still occur, and the optical torque changes its direction from 2-I to a 3-I, the same as that for spherical Ag NPs (Supplementary Figure 16a).

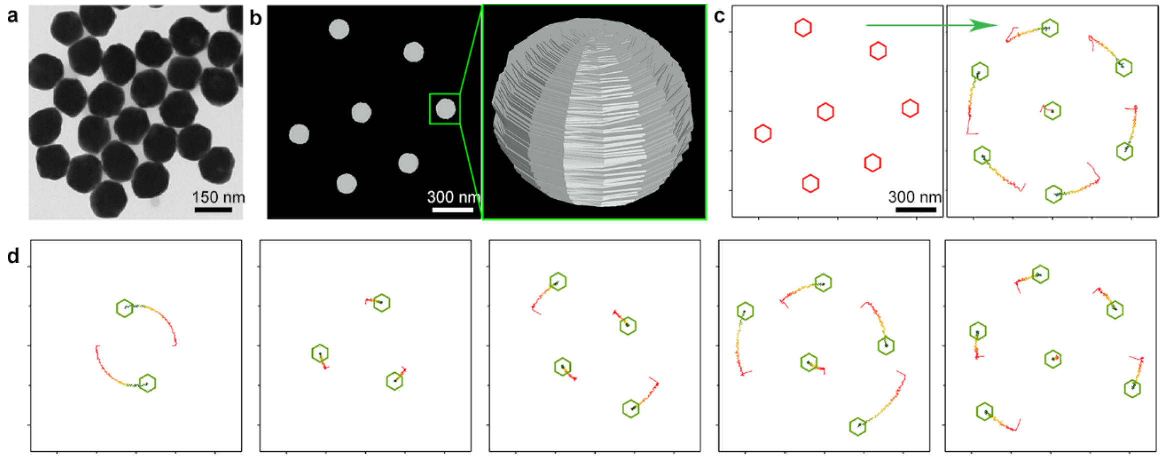

Supplementary Figure 20. Irregular Ag NPs in optical fields. **a**, TEM images of the Ag NPs used in the experiments. Small deviations from perfect spherical shape due to flat facets are observed. **b**, 2D projection and 3D view of irregular Ag NP models used in the simulation. **c**, Simulations of light-driven self-organization and orbital motions of 7 irregular Ag NPs without surface charges in a circularly polarized plane wave. **d**, Orbital motions of 2-6 irregular Ag NPs without surface charges where optical torque reversal happens for optical matter arrays with 3 or more NPs, the same as that for spherical Ag NPs.

Supplementary Note 10. Rotational symmetry and optical torque in the point-dipole approximation

To demonstrate the importance of both symmetry and phase-retardation we performed calculations in the point-dipole approximation. We restrict the treatment to "first-order" scattering both to allow an intuitive understanding of the origin of torque, but also because of the contrasting results that arise when finite particle size is considered in the next part of this discussion. Following from[6], the optical torque on a pair (dimer) of spherical particles A and B in the plane transverse to the propagation of a circularly polarized (left-handed) plane-wave (in water with $\lambda = 800\ nm$ and $150nm$ Ag NP's) is

$$\langle \tau_z \rangle = \sum_{n=\mathrm{A,B}} \mathbf{r}_i \times \mathbf{F}^n = R\frac{|E_0|^2|\alpha_0|^2}{2} Re\left\{ \mathrm{i}\,\alpha_0^*\left(G_{xx}^{\mathrm{AB}*} - G_{yy}^{\mathrm{AB}*}\right)\frac{\partial}{\partial y}\left(G_{xy}^{\mathrm{AB}}\right)\Big|_{r=r_{\mathrm{B}}} \right\} \tag{19}$$

where R is the separation between the particles, E_0 is the magnitude of the electric field, α_0 is the polarizability of the particles, G_{ij}^{AB} are the elements of the dyadic Green's function, and * means complex conjugate. The black curve in Supplementary Figure 21 shows the torque on the dimer as a function of separation. At first order in the point dipole approximation, the torque is always positive and decays as a function of separation. Since the dimer will be driven to rotate in the same direction as the electric field, this is positive torque.

Other symmetries of optical matter arrays are possible for three or more point-dipole particles. We calculated the torque on both two-fold and three-fold symmetric clusters of three particles (trimer) at first order in the point dipole approximation. With $G_{ij}^{\mathrm{AB}} + G_{ij}^{\mathrm{AC}} = f_{ij}$ the torque on a two-fold symmetric trimer is

$$\langle \tau_z \rangle = \sum_{n=\mathrm{A,B,C}} \mathbf{r}_i \times \mathbf{F}^n = 2R\frac{|E_0|^2|\alpha_0|^2}{2} Re\left\{ \mathrm{i}\,\alpha_0^*\left(f_{xx}^* - f_{yy}^*\right)\frac{\partial}{\partial y}\left(f_{xy}\right)\Big|_{r=r_{\mathrm{A}}} \right\} \tag{20}$$

The red curve in Supplementary Figure 21 shows the torque on the two-fold rotationally symmetric trimer as a function of separation. The torque is in the same sense as the dimer. A slight oscillatory character is present in this configuration even for first-order scattering. This is because $R_{\mathrm{AB}} \neq R_{\mathrm{AC}}$ and the relative phase of the scattered light at particle A from particles B and C will generally be different.

For the three-fold symmetric trimer the torque at first order in the point dipole approximation is

$$\langle \tau_z \rangle = \sum_{n=\mathrm{A,B,C}} \mathbf{r}_i \times \mathbf{F}^n = -\sqrt{3}R\frac{|E_0|^2|\alpha_0|^2}{2} Re\left\{ \mathrm{i}\,\alpha_0^*\left(f_{xx}^* - f_{yy}^*\right)\frac{\partial}{\partial x}\left(f_{xy}\right)\Big|_{r=r_{\mathrm{A}}} \right\} \tag{21}$$

The blue curve in Supplementary Figure 21 shows torque on the three-fold rotationally symmetric trimer as a function of separation. The torque is in the opposite direction (i.e. exhibits negative torque) compared to the two-fold symmetric structures.

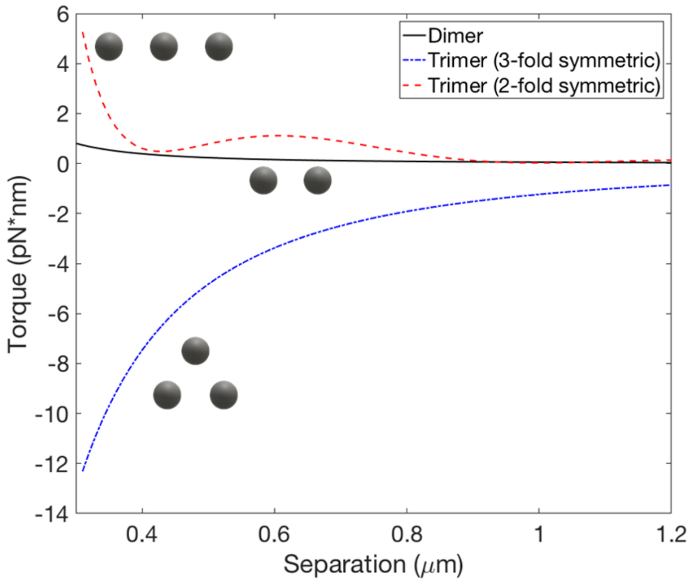

Supplementary Figure 21. Consideration of symmetry and separation on torque of optical arrays. The torque of a nanoparticle dimer (black) and trimers with two (red) and three-fold (blue) rotational symmetry calculated in the point-dipole approximation (Supplementary Equations 19-21). The calculations assumed left-handed circularly polarized plane-wave illumination and only first-order scattering. Each NP configuration is shown schematically.

Supplementary Note 11. Lattice plasmon peak positions in optical matter arrays

The role of symmetry enters through the structure factor of an extended NP array, which depends on the type of lattice[7,8]. The resonant peaks of the lattice plasmon modes for trigonal lattices are given by[8]

$$\lambda_{LP} = \Delta\left[\frac{4}{3}(i^2+ij+j^2)\right]^{-\frac{1}{2}}\left(\frac{\varepsilon_{Ag}(\lambda_{LP})\varepsilon_b}{\varepsilon_{Ag}(\lambda_{LP})+\varepsilon_b}\right)^{\frac{1}{2}} \tag{22}$$

where λ_{LP} is the LP resonant wavelength, Δ is the lattice spacing, $\varepsilon_{Ag}(\lambda_{LP})$ is silver's permittivity function, ε_b is the permittivity of the medium, and i and j are integers that enumerate different LP modes. Supplementary Figure 22 shows the dependence of the resonant wavelength, λ_{LP}, of the LP mode on the lattice spacing, Δ, for $i = 0,\ j = 1$ (01 mode) and $i = 1,\ j = 1$ (11 mode). At a lattice spacing of 600 nm, both modes have resonances that correspond to the two scattering peaks in Fig. 5d (main text). At a wavelength of 800 nm, only the 01 mode has a resonance that corresponds to the 647 nm lattice spacing scattering peak in Fig. 5c (main text).

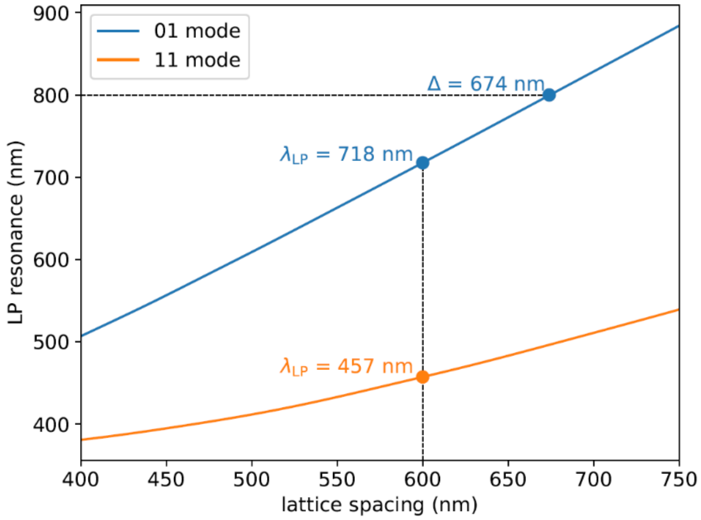

Supplementary Figure 22. Dependence of the lattice plasmon (LP) resonance on lattice spacing for 01 and 11 modes for an infinitely extended optical matter array built on an equilateral trigonal lattice with variable lattice spacing. A lattice spacing of 600 nm (for 150 nm dia. Ag NPs in water) gives LP resonances at 457 nm and 718 nm. Conversely, an 800 nm LP resonance occurs for lattice spacing of 674 nm.

Supplementary References:

1 Figliozzi, P. *et al.* Driven optical matter: Dynamics of electrodynamically coupled nanoparticles in an optical ring vortex. *Phys. Rev. E* **95**, 022604 (2017).

2 Pinchuk, A. O. Size-dependent hamaker constant for silver nanoparticles. *J. Phys. Chem. C* **116**, 20099-20102 (2012).

3 Gargiulo, J. *et al.* Understanding and reducing photothermal forces for the fabrication of au nanoparticle dimers by optical printing. *Nano Lett.* **17**, 5747-5755 (2017).

4 Donner, J. S., Baffou, G., McCloskey, D. & Quidant, R. Plasmon-assisted optofluidics. *ACS Nano* **5**, 5457-5462 (2011).

5 Piazza, R. & Parola, A. Thermophoresis in colloidal suspensions. *J. Phys.: Condens. Matter.* **20**, 153102 (2008).

6 Dholakia, K. & Zemánek, P. Colloquium: gripped by light: optical binding. *Rev. Mod. Phys.* **82**, 1767 (2010).

7 Humphrey, A. D. & Barnes, W. L. Plasmonic surface lattice resonances on arrays of different lattice symmetry. *Phys. Rev. B* **90**, 075404 (2014).

8 Thio, T., Ghaemi, H. F., Lezec, H. J., Wolff, P. A. & Ebbesen, T. W. Surface-plasmon-enhanced transmission through hole arrays in Cr films. *J. Opt. Soc. Am. B* **16**, 1743-1748 (1999).
