# Supplementary material for: Crossover from positive to negative optical torque in mesoscale optical matter
Source: Nat Commun. 2018 Nov 20;9:4897. doi: 10.1038/s41467-018-07376-7 (PMC6244235; doi:10.1038/s41467-018-07376-7)
Supplement: Supplementary file 3 — Description of Additional Supplementary Files [file 41467_2018_7376_MOESM3_ESM.pdf]

Description of Additional Supplementary Files

File Name: Supplementary Movie 1

Description: A montage of 12 movie clips edited to play as a single video showing optical matter arrays with 2-13 silver nanoparticles (150 nm diameter) in water. All movie clips were taken with the same experimental conditions using an optical trapping system at Clarkson University. The laser beam (λ = 800 nm) was left-handed circularly polarized. The frame rate is reduced from 300 fps to 60 fps for better viewing. The rotation direction changes from counterclockwise for arrays with 2-7 nanoparticles to clockwise for arrays with 9-13 nanoparticles while the 8-nanoparticle array is nearly stationary.

File Name: Supplementary Movie 2

Description: A single continuously recorded movie showing assembly of optical matter arrays with 2-13 silver nanoparticles (150 nm diameter) in water. The movie was taken in an independent experiment using an optical trapping system at the University of Chicago. The laser beam (λ = 800 nm) was right-handed circularly polarized. The frame rate is reduced from 100 fps to 50 fps for better viewing. The rotation direction is clockwise for small arrays (e.g., 2 nanoparticles) and counterclockwise for larger arrays (e.g., 9-13 nanoparticles).

File Name: Supplementary Movie 3

Description: A single and continuous movie for a 7- nanoparticle optical matter array with cetyl trimethylammonium bromide added in the solution. The laser beam (λ = 800 nm) was left-handed circularly polarized. The frame rate is reduced from 200 fps to 60 fps for better viewing
